# Supplementary material for: A Novel Structure Harboring blaCTX-M-27 on IncF Plasmids in Escherichia coli Isolated from Swine in China
Source: Antibiotics (Basel). 2021 Apr 4;10(4):387. doi: 10.3390/antibiotics10040387 (PMC8065532; doi:10.3390/antibiotics10040387)
Supplement: Supplementary file 1 [file antibiotics-10-00387-s001.pdf]

Supplementary Data

Table S1. Antimicrobial resistance of 32 CTX-M-27 producing *Escherichia coli* from swine in china [a].

Strain	Antimicrobial agents [b]
1-8	AMP, CTF, CTX, FLF, STR, APR, CIP, NAL, DF, TET, ENR
9-4	AMP, CTF, CTX, STR, APR, CIP, NAL, TET, ENR
3-3	AMP, CTF, CTX, STR, APR, CIP, NAL, DF, TET, ENR
1-12	AMP, CTF, CTX, FLF, CHL, GEN, APR, CIP, NAL, CL, DF, TET, ENR, KAN
3-5	AMP, CTX, FLF, CHL, GEN, APR, CIP, NAL, CL, DF, TET, ENR, KAN
B1-3	AMP, CTF, CTX, FLF, CHL, GEN, CIP, NAL, CL, DF, TET, ENR, KAN
5-15	AMP, CTF, CTX, FLF, CHL, GEN, CIP, NAL, CL, DF, TET, ENR, KAN
B1-13	AMP, CTF, CTX, FLF, CHL, GEN, CIP, NAL, CL, DF, TET, ENR, KAN
B6-5	AMP, CTF, CTX, FLF, CHL, GEN, CIP, NAL, CL, DF, TET, ENR, KAN
B8-11	AMP, CTF, CTX, FLF, CHL, GEN, CIP, NAL, CL, DF, TET, ENR, KAN
M8-1	AMP, CTF, CTX, FLF, CHL, GEN, CIP, NAL, CL, DF, TET, ENR, KAN
6-8	AMP, CTF, CTX, GEN, APR, NAL, ENR

1-3	AMP, CTF, CTX, STR, CIP, NAL, DF, TET, ENR, KAN
1-4	AMP, CTF, CTX, FLF, CHL, STR, APR, NAL, TET, ENR
2-3	AMP, CTF, CTX, STR, CIP, NAL, TET, ENR
5-7	AMP, CTF, CTX, STR, CIP, NAL, TET, ENR
3-13	AMP, CTF, CTX, STR, APR, CIP, NAL, DF, OQX, TET, ENR
1-2	AMP, CTF, CTX, STR, APR, CIP, NAL, DF, OQX, TET, ENR
4-11	AMP, CTF, CTX, CTZ, STR, APR, CIP, NAL, DF, TET, ENR
5-14	AMP, CTF, CTX, STR, AMI, APR, CIP, NAL, DF, OQX, TET, ENR
5-7(2)	AMP, CTF, CTX, STR, CTZ, CIP, NAL, DF, OQX, TET, ENR
3-10	AMP, CTF, CTX, STR, CIP, NAL, DF, OQX, TET, ENR
1-6	AMP, CTF, CTX, STR, CTZ, CIP, NAL, DF, OQX, TET, ENR
3-16	AMP, CTF, CTX, STR, APR, CIP, NAL, DF, TET, FOS, ENR
B8-9	AMP, CTF, CTX, STR, CTZ, CIP, NAL, DF, OQX, TET, ENR
5-10	AMP, CTF, CTX, STR, APR, CIP, NAL, DF, TET, ENR
4-4	AMP, CTF, CTX, FLF, CHL, GEN, APR, CIP, NAL, CL, DF, TET, ENR, KAN
6-9	AMP, CTF, CTX, STR, APR, CIP, NAL, TET

M5-2	AMP, CTF, CTX, FLF, STR, APR, CIP, NAL, DF, TET, ENR
5-1	AMP, CTF, CTX, STR, CIP, NAL, DF, OQX, TET, ENR
4-9	AMP, CTF, CTX, STR, APR, CIP, NAL, DF, TET, ENR
5-18	AMP, CTF, CTX, STR, APR, CIP, NAL, DF, TET, ENR

All of the 32 isolates were multidrug-resistant (MDR) strains, being resistant to at least seven antibiotics.

a: The antibiotics susceptibility test of CTX-M-27-producing *E.coli* were determined using the agar dilution method and the microdilution broth method (Colistin). The results were interpreted following Clinical and Laboratory Standards Institution (CLSI) guidelines (2015, M100-S25) and veterinary CLSI (VET01-A4/VET01-S2).

b: AMP: Ampicillin; CTX: Cefotaxime; CTF: Ceftiofur; CTZ: Ceftazidime; CIP: Ciprofloxacin; ENR: Enrofloxacin; KAN: Kanamycin; GEN: Gentamicin; AMI: Amikacin; TET: Tetracycline; CHL: Chloramphenicol; FLF: Florfenicol; CL: Colistin; STR: Streptomycin; APR: Apramycin; NAL: Nalidixic acid; DF: Dafloxacin; OQX: Olaquindox; FOS: Fosfomycin.
